# A comparison in women with newly diagnosed diabetes between those with and without a history of gestational diabetes: a new perspective

**DOI:** 10.1007/s00592-023-02096-x

**Published:** 2023-04-29

**Authors:** Anna Stogianni, Eva Melin, Jonathan Dereke, Mattias Rööst, Magnus Hillman, Mona Landin-Olsson, Pär Wanby, Maria Thunander

**Affiliations:** 1 Department of Clinical Sciences, Endocrinology and Diabetes, Lund University, Lund, Sweden; 2 Department of Endocrinology, Metabolism and Diabetes, Karolinska University Hospital, Huddinge, Stockholm, Sweden; 3 Department of Research and Development, Region Kronoberg, Växjö, Sweden; 4 Primary Care, Region Kronoberg, Växjö, Sweden; 5 Diabetes Laboratory, Biomedical Center, Lund University, Lund, Sweden; 6 Department of Clinical Sciences, Family Medicine, Lund University, Malmö, Sweden; 7 Department of Diabetology and Endocrinology, Skane University Hospital, Lund, Sweden; 8 Institution of Health and Optometry, Linnaeus University, Kalmar-Växjö, Sweden; 9 Department of Internal Medicine, Endocrinology and Diabetes, Kalmar County Hospital, Region Kalmar, Kalmar, Sweden; 10 Department of Internal Medicine, Endocrinology and Diabetes, Växjö Central Hospital, Växjö, Sweden

**Keywords:** Gestational diabetes, Type 2 diabetes, Type 1 diabetes, Cardiovascular disease, Hypertension, C-peptide, GADA

## Abstract

**Aims:**

Previous gestational diabetes mellitus (GDM) entails increased risk of future diabetes. We describe the characteristics of women with previous GDM and compare with no previous GDM from the cohort Diabetes in Kalmar and Kronoberg (DKK) of 1248 adults, 40% women, with new diabetes, and factors affecting age and C-peptide levels at diagnosis of diabetes.

**Methods:**

Age-at-diagnosis of diabetes, BMI, hypertension, hyperlipidemia, smoking, physical activity, and pre-existing myocardial infarction, stroke, or peripheral arterial insufficiency were registered at ordinary care visits close to diagnosis of diabetes, for the 43 women (9.4% of 456 from DKK with complete data for this analysis) with self-reported previous GDM (yes/no) and 86 controls without it, matched for date of diagnosis of diabetes. Blood samples were centrally analyzed for GADA and C-peptide for classification of diabetes.

**Results:**

Women with previous GDM had lower mean age-at-diagnosis of diabetes, 53.4 vs 65.0 years, lower systolic blood pressure (SBP), 131.2 vs 137.5 mmHg, and fewer had pre-existing hypertension than without previous GDM (*p* < 0.001–0.05). Among antibody negative women with previous GDM, BMI (*p* = 0.024), hypertension (*p* = 0.023) and hyperlipidemia (*p* < 0.001) were associated with higher levels of C-peptide, while physical activity was inversely associated (*p* = 0.035), and SBP (*p* = 0.02) and hypertension (*p* = 0.016) were associated with age-at-diagnosis of diabetes.

**Conclusions:**

Women with previous GDM were a decade younger and had lower prevalence of hypertension at diagnosis of diabetes; C-peptide levels were associated with BMI, hypertension, and hyperlipidemia and showed a tendency to be lower, possibly indicating a phenotype with higher risk of overt cardiovascular disease later in life.

## Introduction

Gestational diabetes mellitus is a state of glucose intolerance with onset during pregnancy [1]. Pregnancy creates a state of insulin resistance due to the production of placental hormones, and thus, pregnancy itself provides a natural “stress test” for diabetes risk [2]. A gradual increase in the prevalence of gestational diabetes has been observed worldwide [3]. Although genetic predisposition establishes susceptibility, environmental factors like the modern lifestyle with increased prevalence of obesity, reduced physical activity, and smoking are the most probable explanations for the increased prevalence of most diabetes types [4]. The risk of developing type 2 diabetes after gestational diabetes is described to increase linearly with the duration of follow-up after pregnancy, but studies vary in their estimates of risk [5–7].

Despite this strong connection we found no studies comparing women with newly diagnosed adult-onset diabetes, with and without a known history of gestational diabetes, only one that prospectively investigated for 15 years women at risk [8]. We therefore turned the perspective and compared women with previous gestational diabetes who developed diabetes with those with newly diagnosed adult-onset diabetes without previously known gestational diabetes.

## Aim

The primary aim of this study was to identify women with newly diagnosed type 2 or type 1 diabetes who had a known history of gestational diabetes and describe their metabolic and lifestyle characteristics at the time of diagnosis of diabetes in a recent cohort of adult patients from southeastern Sweden with newly diagnosed type 1 or type 2 diabetes, and to compare with characteristics of women with new diabetes without previously known gestational diabetes from the same cohort. Factors affecting age and C-peptide levels at diagnosis of diabetes in these women were also explored.

## Subjects and methods

### Population

This study included data from the Swedish observational study Diabetes in Kronoberg and Kalmar (DKK) that included patients with newly diagnosed adult-onset diabetes (ages 18–100 years) during 2016–2017, including 1248 patients [9]. Five hospitals (100%) and 55 (83%) Primary Health Care Centers (public and private) in Kronoberg and Kalmar counties, with together a population of 433 000 inhabitants, in southeastern Sweden, invited and included patients consecutively during routine care visits, acute, and scheduled. According to electronic records data 50% of the adult patients with new diabetes during the period were included. Inclusion criteria were adults ≥ 18 years with newly diagnosed (most within 3, and up to 14, the absolute majority within 100 days) type 1 or type 2 diabetes 2016–2017, residing in Kronoberg or Kalmar. Exclusion criteria were current gestational diabetes or incomplete data for this analysis (n = 107), men (n = 685, 60%) leaving 456 women (40%), of whom all 43 women with a known history of previous gestational diabetes were included (9.4%). For comparison 86 women from the DKK cohort with new diabetes but without a known history of gestational diabetes, matched (2:1) for date of diagnosis of diabetes, were investigated, meaning 28% (n = 129) of the women were included in this study. DKK was approved by the Ethical Review Board of Linköping University (DNR 2015–350-31, 2017–354-32). All participants gave written informed consent prior to collection of any data.

### Examinations and laboratory procedures

Data regarding age-at-diagnosis of diabetes (years), weight (kg), length (cm), BMI (kg/m^2^), waist circumference (cm), ethnic background (Caucasian, Asian, African), born in Sweden (yes/no), tobacco use (never, ex user, user), moderate exercise equivalent to ≥ 3 times/week were reference to 1–2 times/week or 0 = inactive, systolic and diastolic blood pressure (SBP, DBP) (standardized procedure, mmHg), and prevalence of hypertension and other pre-existing cardiovascular conditions, as previous myocardial infarction/ischemic heart disease, stroke/TIA, and peripheral arterial insufficiency (clinical diagnosis, always based on standardized BP measurements at several levels of lower extremities) were collected according to a standard questionnaire via interview by healthcare professional, who also had access to the patient´s electronic records since 2005. Also included were the prevalence of previous depression (clinical diagnosis and/or use of antidepressants), thyroid disorder, and family history of diabetes.

Blood samples were collected and transported according to standard procedures, to the laboratories of the departments of clinical chemistry in the regional hospitals, then the extra samples, also with standardized procedures, to the diabetes research laboratory at Skane/Lund University Hospital where they were centrifuged, aliquoted, and frozen to minus 80 degrees, for further analyses of islet antibodies and C-peptide.

Analyses of lipid levels (T-cholesterol, triglycerides, LDL and HDL, mmol/l), and venous HbA1c (mmol/mol) were performed according to clinical routine at the clinical chemistry laboratories of the participating hospitals, as were a venous p-glucose collected simultaneously with the C-peptide samples (random, for best estimate of beta-cell function) [9, 10]. GADA (glutamic acid decarboxylase antibodies) IA-2A and ZnT8A were analyzed with enzyme-linked immunosorbent assay (ELISA) (RSR Ltd, Cardiff, UK) according to manufacturer’s instructions. C-peptide levels were analyzed with ELISA (Mercodia, Uppsala, Sweden) according to manufacturer’s instructions, detection limit 25 pmol/L [9].

### Definitions and classification of diabetes and the metabolic syndrome

The diagnosis of diabetes was confirmed if fasting plasma glucose was ≥ 7.0 mmol/L at least twice; or one random plasma glucose ≥ 11.1 mmol/L (venous) or ≥ 12.2 mmol/L (capillary); or one post-load plasma glucose ≥ 11.1 mmol/L (venous) or ≥ 12.2 mmol/L (capillary) after a 75 g oral glucose tolerance test (OGTT); or HbA1c ≥ 48 mmol/mol and fp-glucose ≥ 6.5 mmol/l; in accordance with ADA and WHO guidelines for diagnosis and classification of diabetes [1, 11].

Patients positive to any islet antibody (GADA; if C-peptide < 0.50 nmol/l IA-2A (tyrosine phosphatase) and ZnT8 (zinc transporter 8) antibodies were also analyzed), or with C-peptide < 0.25 nmol/L, was classified as type 1 diabetes; islet antibody-negative patients with C-peptide ≥ 0.25 nmol/L were classified as type 2 diabetes [12–14].

The metabolic syndrome was defined by the presence of central adiposity defined as waist circumference ≥ 88 cm or BMI > 30 kg/m^2^ plus two or more of the following four factors 1) triglycerides ≥ 1.7 mmol/l (≥ 150 mg/dl) or specific treatment for this lipid abnormality; 2) HDL cholesterol < 1.29 mmol/l (< 50 mg/dl) in women or treatment for this lipid abnormality; 3) systolic blood pressure ≥ 130 mm Hg or diastolic blood pressure ≥ 85 mm Hg or treatment for previously diagnosed hypertension; and 4) “IGT or diabetes,” and all participants in this study by definition had diabetes, since it was an inclusion criterium [15].

### Statistical methods

Descriptive data if normally distributed were described by mean and standard deviation. Number and percentage are reported for categorical variables. Differences between groups were evaluated with Chi-square test (categorical) and Student´s unpaired T test (continuous variables). For estimation of linear associations, simple linear regression analyses were performed for the dependent variables age-at-diagnosis of diabetes and C-peptide level*.* Variables with *p*-values < 0.25 were included in multiple regression models (stepwise backward Wald). Independent variables in each multiple regression can be derived from the tables, or the descriptions in the results section. Characteristics and differences between women with/without previous gestational diabetes were analyzed for all, and for GADA-negative/positive. All tests were two-tailed. *P*-value ≤ 0.05 was considered significant. All statistical analyses were performed with SPSS (Statistical Package for the Social Sciences, Chicago, Illinois) version 23.

## Results

There were 43 women with previous gestational diabetes and 86 controls without previously known gestational diabetes. The 43 women with previous gestational diabetes were 9.4% of all women in DKK, where 40% of patients with new diabetes were women. Characteristics for both groups of women are displayed in Table 1. Most of the women with previous gestational diabetes and new adult-onset diabetes had type 2 diabetes (*n* = 37, 91%). Six had type 1/LADA (9%).

**Table 1:** Characteristics of women with new diabetes in Kalmar and Kronoberg 2016–2017, a comparison between those with and without a history of gestational diabetes

	Women with a history of gestational diabetes		Women without a history of gestational diabetes		*P*	*P*
*N*	All 43	GADA neg 37	All 86	GADA neg 78	All	GAD neg
*Basal characteristics*						
Age (years)	53.4 ± 13.9	54.3 ± 14.1	65 ± 10.9	65.1 ± 10.8	< 0.001	< 0.001
Weight (kg)	82.8 ± 16.5	84.6 ± 16.5	85.4 ± 15.1	86.1 ± 14.4	0.39	0.61
Length (cm)	160.3 ± 25.9	159.7 ± 27.4	158.5 ± 25.2	157.9 ± 26.3	0.70	0.74
BMI (kg/m^2^)	30.9 ± 6	31.4 ± 6	32.3 ± 5.6	32.6 ± 5.4	0.24	0.29
Waist circumference (cm)	102.1 ± 15.4	103.7 ± 14.9	104 ± 11.6	105 ± 10.1	0.44	0.60
Caucasian	41 (95%)	35 (94.6%)	82 (95%)	74 (96.1%)	0.14	0.13
Born in Sweden	34 (79%)	29 (80.6%)	68 (79%)	60 (77%)	0.80	0.81
Smoking	9 (21%)	7 (18.9%)	10 (12%)	8 (10%)	0.12	0.26
Physical activity ≥ 3 times/week	20 (47%)	18 (48.7%)	45 (52%)	41 (53.3%)	0.49	0.65
SBP (mmHg)	131.2 ± 16.9	131.4 ± 16.8	137.5 ± 16.5	137.7 ± 16.6	0.047	0.06
DBP (mmHg)	79.2 ± 10.1	78.9 ± 9.7	79 ± 10.9	79.2 ± 10.9	0.93	0.88
*Pre-existing conditions*						
Hypertension	17 (40%)	15 (43%)	52 (61%)	47 (60.3%)	0.044	0.086
Hyperlipidemia	15 (35%)	14 (41.2%)	32 (37%)	31 (40.3%)	0.99	0.93
Myocardial ischemia	1 (2.3%)	1 (3%)	6 (7%)	5 (6.8%)	0.30	0.436
Stroke/TIA	1 (2.3%)	1 (3%)	7 (8.1%)	7 (9.8%)	0.22	0.25
Arterial insufficiency	0 (0%)	0 (0%)	1 (1.2%)	0 (0%)	0.51	ns
Previous depression	11 (26%)	10 (29%)	24 (28%)	20 (26%)	0.87	0.77
Metabolic syndrome	36 (84%)	31 (86%)	76 (88%)	70 (90%)	0.78	0.57
Thyroid disorder	6 (14%)	6 (18.2%)	12 (14%)	11 (14.1%)	0.87	0.59
Family history T1D	6 (14%)	3 (9.1%)	5 (5.8%)	4 (5.1%)	0.08	0.43
Family history T2D	26 (61%)	21 (64%)	49 (57%)	45 (58%)	0.34	0.61
*Laboratory data*						
HbA1c (mmol/mol)	64.3 ± 24.5	62.8 ± 23.7	58.2 ± 23	56.8 ± 20.4	0.16	0.17
Tot cholesterol (mmol/l)	5.9 ± 1.2	5.9 ± 1.2	5.6 ± 1.7	5.6 ± 1.6	0.25	0.35
LDL (mmol/l)	3.8 ± 0.9	3.9 ± 0.9	3.5 ± 1.4	3.6 ± 1.3	0.25	0.25
HDL (mmol/l)	1.3 ± 0.5	1.3 ± 0.5	1.3 ± 0.5	1.3 ± 0.5	0.86	0.98
TG (mmol/l)	2.3 ± 1.4	2.2 ± 1.3	2.0 ± 0.9	2.0 ± 0.9	0.14	0.21
C peptide (nmol/l)	1.05 ± 0.63	1.1 ± 0.64	1.3 ± 0.7	1.3 ± 0.6	0.07	0.098

**Fig. 1:**
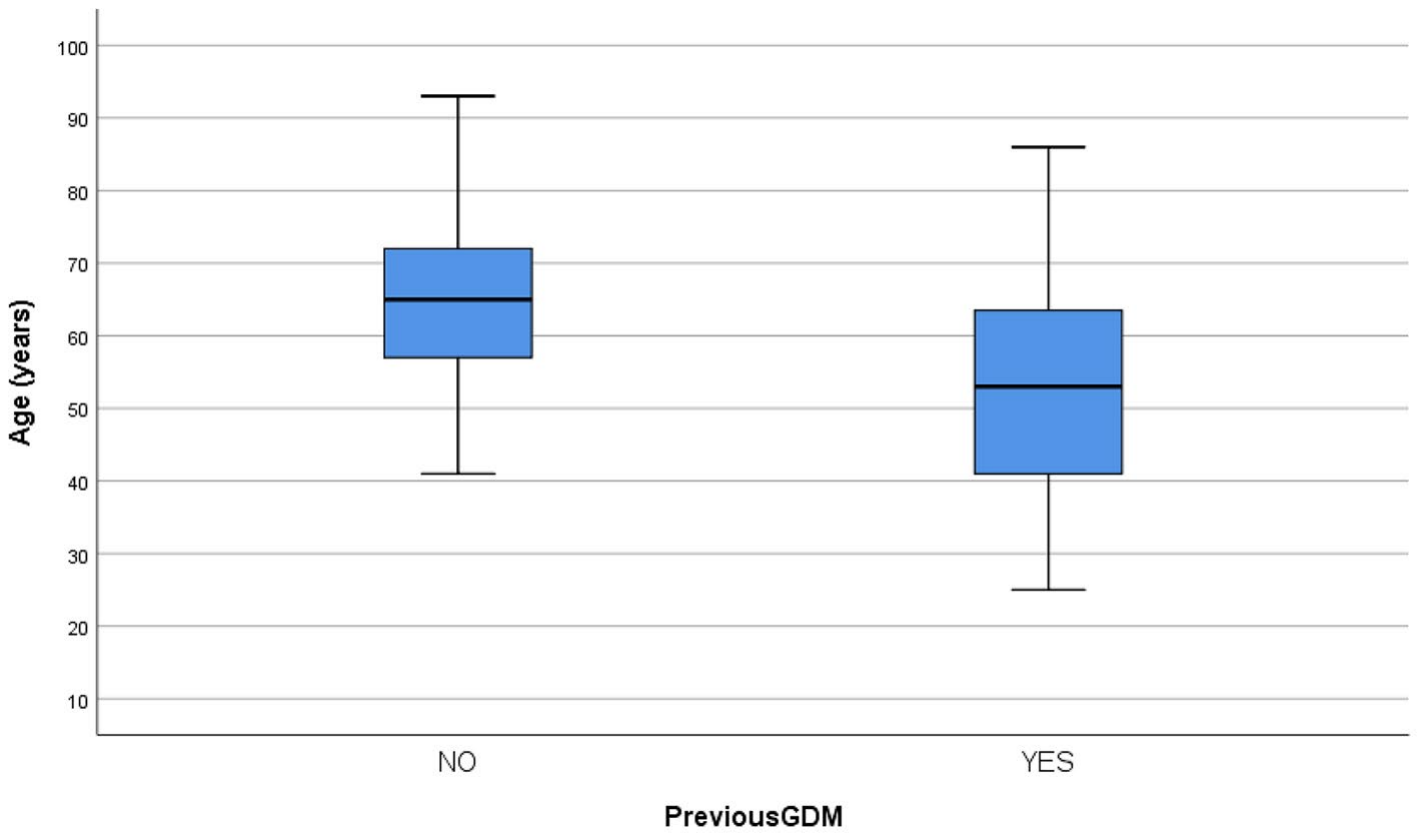
Age distribution in women with new diabetes with or without a history of gestational diabetes in Kalmar and Kronoberg 2016–2017. *P =  < 0.001

Women with previous gestational diabetes had lower mean age at diagnosis of type 2 diabetes than those without previous gestational diabetes 53.4 ± 14 vs 65.0 ± 11 years (*p* < 0.001) (Fig. 1). Their SBP was lower 131.2 ± 17 vs 137.5 ± 16.5 mm Hg (*p* = 0.047), and fewer had hypertension before diagnosis, 39.5% vs 60.5% (*p* = 0.044). Women with previous gestational diabetes tended to have a family history of T1D more often, (14%), in comparison with those without previous gestational diabetes (5.8%) (*p* = 0.08), but significance was lost when excluding all women that were GADA positive.

There were no differences, however, between the two groups regarding risk factors as BMI, waist circumference, hyperlipidemia, smoking, or prevalence of the metabolic syndrome as a whole. Neither were there any differences in prevalence of pre-existing cardiovascular conditions as myocardial infarction, stroke, or peripheral arterial insufficiency. There was a trend that beta cell function measured by random C-peptide was lower in women with previous gestational diabetes in comparison with those without, 1.05 ± 0.63 vs 1.27 ± 0.65 nmol/l (*p* = 0.07) and a weak tendency persisted when excluding women with GADA positivity (*p* = 0.098). There were no differences between previous/no previous gestational diabetes and/or GADA negative/positive patients regarding levels of simultaneous, sp-glucose or HbA1c, *p* = ns for all, except for in the GADA negative women mean sp-glucose was 8.7 ± 4.1 (no previous GDM) vs 10.6 ± 4.9 (previous GDM), *p* = 0.064.

Levels of HbA1c, total cholesterol, LDL and TG, at diagnosis of diabetes, were slightly higher in the group of women with previous gestational diabetes, but the differences did not reach significance.

SBP (*p* = 0.002) and previous hypertension (*p* = 0.03) were associated with age-at-diagnosis of diabetes among women with a history of gestational diabetes. Higher BMI also tended to be associated with earlier onset of diabetes (*p* = 0.066) in women with previous gestational diabetes. In multiple regression analysis among women with previous gestational diabetes SBP (*p* = 0.035) remained associated with the outcome age-at-diagnosis, but BMI (*p* = 0.06) and pre-existing hypertension (*p* = 0.09) lost their significance when adjusting for SBP, BMI, and pre-existing hypertension.

In the group of women without previous gestational diabetes, earlier onset of diabetes was also associated with SBP (*p* = 0.027) and previous hypertension (*p* = 0.02) (Table 2). In addition, higher BMI, waist circumference, and DBP were associated with earlier diagnosis of diabetes in the group without previous gestational diabetes.

**Table 2:** Factors affecting age and C-peptide levels at diagnosis of diabetes in women with new diabetes with or without a history of gestational diabetes in Kalmar and Kronoberg 2016–2017

	Women with a history of gestational diabetes		Women without a history of gestational diabetes	
	*P* (All)	*P* (GADA neg)	*P* (All)	*P* (GADA neg)
*Age at diagnosis*				
BMI (kg/m^2^)	0.066	0.046	< 0.001	< 0.001
Waist circumference (cm)	ns	ns	0.001	0.001
SBP (mmHg)	0.002	0.016	0.027	0.011
DBP (mmHg)	ns	ns	0.003	0.008
HbA1c (mmol/mol)	ns	ns	0.052	ns
C-peptide (nmol/l)	0.077	0.19	ns	ns
Pre-existing hypertension	0.03	0.021	0.02	0.019
*C-peptide*				
Age	0.077	0.19	ns	ns
BMI (kg/m^2^)	0.016	0.024	ns	ns
Waist circumference (cm)	0.039	0.14	0.085	0.19
Physical activity ≥ 3 times/week	0.036	0.035	0.066	0.14
SBP (mmHg)	ns	ns	ns	ns
DBP (mmHg)	ns	ns	0.051	ns
HbA1c (mmol/mol)	ns	ns	ns	ns
Pre-existing hypertension	0.048	0.023	0.017	0.026
Pre-existing hyperlipidemia	0.001	< 0.001	0.02	0.083
Pre-existing stroke	ns	ns	0.005	0.008
Tot cholesterol (mmol/l)	ns	0.084	ns	ns

Simple linear regression analyses were performed between demographic/metabolic variables and age and C-peptide levels at diagnosis of diabetes

Among women with previous gestational diabetes, increased BMI (*p* = 0.016) and waist circumference (*p* = 0.004), pre-existing hypertension (*p* = 0.048), and hyperlipidemia (*p* = 0.001) were associated with higher levels of C-peptide, while physical activity was inversely associated (*p* = 0.036) (Table 2). In multiple regression analysis among women with previous gestational diabetes BMI (*p* = 0.003), pre-existing hyperlipidemia (*p* < 0.0001), and physical activity (*p* = 0.007) remained associated with C-peptide; and age-at-diagnosis (*p* = 0.004) and total cholesterol (*p* = 0.008) became so, while waist circumference and pre-existing hypertension lost their significances (*p* = 0.4–1.0).

In the women without previous gestational diabetes, C-peptide remained associated with risk conditions as pre-existing hypertension (*p* = 0.017) and hyperlipidemia (*p* = 0.02), and with previous stroke (*p* = 0.005), but not with BMI and waist circumference. Here C-peptide level decreased with rising DBP (*p* = 0.051) and tended to decrease with increased physical activity (*p* = 0.066).

In simple regression analyses sp-glucose did not affect level of C-peptide, neither for all, for women pos/neg for GADA, or without previous gestational diabetes, all ns. In multiple regression analysis only previous GDM significantly affected level of C-peptide, *p* = 0.03, while sp-glucose (*p* = 0.91) and GADA pos/neg (*p* = 0.15) did not. The same was true for HbA1c.

## Discussion

In this study we examined women with newly diagnosed adult-onset diabetes who had a history of gestational diabetes and compared with a group of women from the same region and time period with newly diagnosed diabetes but without a known history of gestational diabetes, matched for date of diagnosis of diabetes.

The women with previous gestational diabetes were a decade younger at diagnosis of diabetes than the women without a history of gestational diabetes (Fig. 1). Women with a history of gestational diabetes have a well-established higher risk of type 2 diabetes [5–7]. According to a systematic review from 2002 this risk increased markedly during the first 5 years after delivery and appeared to reach a plateau after 10 years [6]. More recent studies, however, have shown that this progression increases linearly with the duration of follow-up with an estimated risk for type 2 diabetes of around 60% at 50 years postpartum. In our study with a very long retrospective perspective of up to 40–60 years, unlike most studies that usually explore prospectively from pregnancy, the women in the group with previous gestational diabetes had a mean age of 54 ± 14 years at diagnosis indicating a continued risk for development of diabetes even many years after a pregnancy complicated by gestational diabetes, emphasizing the importance of this perspective [7, 16]. The progression to overt diabetes occurred at a younger age in women with previous gestational diabetes in comparison with those without which was in line with a 15-year prospective study which also indicated a shorter median time for developing diabetes in women with a history of gestational diabetes, compared to a control group without gestational diabetes [8]. Mean age of onset in our control group was 65 years, the same as the mean age of diagnosis of type 2 diabetes in our previous population-based incidence study from the same region [13]. In a fifteen-year follow-up of that study the risk of earlier onset type 2 diabetes was clearly demonstrated, as onset before age 40 years carried a 5.6 HR for mortality within 15 years from diagnosis [17].

Among the factors associated with age-of-onset of diabetes in both the present study groups were SBP, previous hypertension, and BMI and in addition DBP in the group without previous gestational diabetes. In a large English cohort study of adults, free of diabetes and cardiovascular disease, both SBP and DBP were continuously related to risk of new-onset diabetes [18]. This suggests that individual and population-based efforts to lower blood pressure, especially targeting individuals with high BMI, may also contribute to lowering the incidence of diabetes.

Having a family history of any type of diabetes was more common in the group with previous gestational diabetes compared to the group without it as was also found in a Swedish study where all included 16 women with diabetes diagnosis and previous gestational diabetes had a first- or second-degree relative with diabetes [19]. In the present study, women with previous gestational diabetes tended to be more likely to have a family history of type 1 diabetes in comparison with those without gestational diabetes, although in both groups most did not. This presence of family history of type 1 diabetes was associated with GADA positivity. In this study 10% of the women with previous gestational diabetes were found to be positive for GADA antibodies showing a much higher prevalence of type 1 diabetes in women after gestational diabetes than in the general population in Denmark (0.5%) [20]. Our finding was in accordance with a study from southern Sweden that found around 8% of women with gestational diabetes positive to islet autoantibodies [21].

The systolic blood pressure tended to be lower in the women with a history of gestational diabetes. A similar observation was reported from the US Diabetes Prevention Program which identified women with IGT and divided them by a reported history of gestational diabetes [22]. In recent years more women with a history of gestational diabetes are being followed and, if needed, treated for hypertension, compared to before. This likely reflects the effect of early behavior-change interventions targeting women with previous gestational diabetes promoting weight loss by change in diet habits, increased physical activity and decreasing sedentary behavior according to the action plan for prevention of diabetes and relative reduction in premature mortality caused by diabetes by 2025 proposed by WHO in 2013 and/or during the follow-up initiated treatment with antihypertensive medication [23].

C-peptide is widely used as a tool for estimation of endogenous insulin secretion [13, 14, 24, 25]. In this study random C-peptide in the GADA negative women showed a tendency, which might possibly have been stronger with a larger sample of women, to be lower in women with new diabetes who were previously affected by gestational diabetes, in comparison with those who were not, indicating a more severe beta-cell dysfunction at diagnosis. This might relate to a predisposition for cardiovascular complications and earlier debut of other long-term complications [26]. Whether sp-glucose, or HbA1c, as marker of longer-time hyperglycemia, affected the levels of C-peptide measured, cannot be completely ruled out, but there were no statistically significant differences between the levels of sp-glucose or HbA1c between the women with/without previous gestational diabetes, neither among GADA positive/negative, and there were few women with very high sp-glucose or HbA1c. Among women with previous gestational diabetes C-peptide was also associated with BMI and pre-existing cardiovascular conditions as hypertension and hyperlipidemia [19]. Taking into consideration that 26% of the women in that group were overweight and 60% obese in addition to the associations mentioned between C-peptide and body composition, lipid status and blood pressure, might indicate that women with gestational diabetes are also at higher risk of overt cardiovascular disease later in life [27]. C-peptide levels were lower with increased physical activity, in line with a recent German study were moderate to vigorous physical activity might have had beneficial effects on insulin and C-peptide metabolism in children and adolescents with a family background of diabetes [28], and logical to physical activity decreasing insulin resistance [29].

Participants with and without a reported history of gestational diabetes were otherwise comparable with respect to risk factors as BMI, waist circumference, hyperlipidemia, smoking and pre-existing myocardial infarction, stroke or peripheral arterial insufficiency. Likewise, metabolic control (HbA1c) and lipid profile at diagnosis did not differ between the groups, indicating that gestational diabetes and type 2 diabetes share common pathogenic mechanisms and risk factors characterized by insulin resistance associated with inadequate insulin secretion [30].

The strengths of our study are first that the perspective is new, looking retrospectively from diagnosis of diabetes to the potential presence of previous gestational diabetes, with a several decades long period of observation. Secondly, our patients were carefully categorized, with analysis of both islet antibodies and C-peptide for correct classification of diabetes type [12, 14]; third, two controls were investigated for each case, matched for date of diagnosis of diabetes. There are also some limitations to our study. Most importantly due to the size of the DKK study, the number of participants in this substudy identified with a previous history of gestational diabetes was only 43, limiting the possibilities to disclose more clear significant associations. On the other hand, there were nevertheless some significant differences, and trends and tendencies, to report, and the work may be hypotheses-generating. Another limitation was that previous gestational diabetes status was self-reported and thus only verified if in the same region the last decades, but practically all pregnant women in Sweden attend antenatal care, where the condition is actively observed for. Further we did not collect information on whether women without a history of gestational diabetes had previous pregnancy. In addition, some mostly elderly women may have been undiagnosed with gestational diabetes during pregnancy because universal testing for gestational diabetes was not standard of care when they were potentially pregnant, and definition of gestational diabetes has also changed over time. This may potentially have led to that a few women with previous undiagnosed gestational diabetes were included in the non-gestational diabetes arm. If anything, this would decrease some of the differences found, and not the opposite.

## Conclusions

In conclusion we found that that the women with previous gestational diabetes were a decade younger and tended to have lower SBP and reduced prevalence of pre-existing hypertension at diagnosis of diabetes compared to women without a history of gestational diabetes. C-peptide levels in women with previous gestational diabetes showed an indication to be lower which might indicate a greater beta-cell dysfunction and were associated with BMI and pre-existing cardiovascular conditions as hypertension and hyperlipidemia, indicating that women with new diabetes that are previously affected by gestational diabetes may be at higher risk of overt cardiovascular disease later in life. The findings indicate similarities, but also differences in the phenotypes of women with especially type 2 diabetes that have previously been affected by gestational diabetes, and those who have not, and these differences warrant further investigation, and clinical consideration.
